# Quackery in Georgia

**Published:** 1900-02

**Authors:** 


### QUACKERY IN GEORGIA.

ON another page will be found a communication from Dr. F. M. Ridley, the President of the Georgia Medical Examining Board (Regular) in reply to a recent editorial of ours on "Quackery in Georgia." We are exceedingly glad to receive this communication, for two reasons. First, because it comes from the president of one of the boards, who explains fully the defects in the law, and explains how illegally practicing physicians may be properly prosecuted; and, in the second place, because it gives THE JOURNAL-RECORD OF MEDICINE an opportunity to do justice to whom justice is due. The article in question was written with malice towards no one, and especially towards the members of the Medical Examining Board (Regular), who are all personal friends of the writer, but was written partly in ignorance of the exact wording of the law, and partly for the purpose of bringing a reply from some member of the board. The latter purpose has been accomplished. If we had told the plain, unvarnished facts of the law, our good friend Dr. Ridley would never have written and explained fully the position of the members of the board and the wording of the law, nor would he have explained how the guilty culprits could be punished. Time and again we have heard it asked why illegally practicing physicians were not prosecuted, but no one could answer, and no one seemed to know what officers of the law there were whose duty it was to prosecute such individuals. We, as well as the physicians throughout the State, are glad to receive this explanation from the President of one of the boards, and we wish to acknowledge here that such criticisms as were contained in the editorial were made in ignorance of the full import of the law, and must therefore be unjust criticism of the members of any of the boards. The law as at present existing is defective, and really, therefore, does not accomplish what it might in regulating the practice of medicine throughout the State. Power should have been given to the President of the board to have cases prosecuted when such should be reported to him as needing the exercise of their provisions. As we have before stated, the hardships fall on the young graduate and the honest practitioner, while the "hardened sinner" knows he is liable to escape prosecution, and will therefore keep shy of the Examining Board. That this is done is evident by the number of such cases in our large cities and even in our rural districts. The law is not only defective in the results accomplished, but it has, in addition, erroneous wording when it says, an act established for "the protection of the people from illegal and unqualified practitioners of medicine and surgery." Of course all this is no fault of the Examining Board, but is a defect in the law itself, and we are only too glad to allow our criticisms to be turned against the law, and not against the Medical Examining Boards. We would like, however, to correct an error in the mind of the President of the Regular Board, and that is, our editorial criticisms were not directed specifically against his board, but against those of the Homeopathic and Eclectic as well. There was no reason for any discrimination to be made. It is a well known fact that all the itinerant quacks who have ever been driven from Atlanta have been done so by the Eclectic Board. A notable instance of this was the celebrated Abbo Institute. It was for this reason that we were led into error in making editorial criticisms, for we very justly thought that if the Eclectic Board had accomplished such excellent results through the legal power vested in it, that the same could be accomplished through all the boards if they felt so disposed. That the law, as it exists at present, needs a radical change must be evident to every medical man, and a determined effort should be made to have this done so as to make it more effective. It is our firm belief that at present the law is a hardship only on the honest physicians who desire to practice in the State. While it is true that every physician in a measure is responsible for the presence of those illegally practicing, it is also true that such seldom has time to investigate the credentials of every newcomer in his midst. As long as the law is in its present shape it will not be effective. If the law cannot be changed, then, to our mind, the best solution of the question is to have a committee appointed from the State Medical Association whose duty it is to have prosecuted those who are offenders of the medical law when such are reported to them; and if it is necessary to employ legal counsel, let such be paid out of the treasury of the State association instead of yearly furnishing embossed stationery for every officer and committeeman in the association. R.

